# Mendelian randomization of risk factors for premenstrual disorders

**DOI:** 10.1038/s44184-026-00231-4

**Published:** 2026-07-16

**Authors:** Yihui Yang, Bowen Tang, Elgeta Hysaj, Nanyan Xiang, Susu Qu, Sara Hägg, Unnur A. Valdimarsdóttir, Elizabeth Bertone-Johnson, Yi Lu, Donghao Lu

**Affiliations:** 1 Institute of Environmental Medicine, Karolinska Institutet, Stockholm, Sweden; 2 Health Management Center, General Practice Medical Center, Lab of Health Data Science, Innovation Institute for Integration of Medicine and Engineering, Frontiers Science Center for Disease-Related Molecular Network, West China Hospital, Sichuan University, Chengdu, Sichuan China; 3 Department of Medical Epidemiology and Biostatistics, Karolinska Institutet, Stockholm, Sweden; 4 Center of Public Health Sciences, Faculty of Medicine, University of Iceland, Reykjavík, Iceland; 5 Department of Epidemiology, Harvard T.H. Chan School of Public Health, Boston, MA USA; 6 Department of Biostatistics and Epidemiology, School of Public Health and Health Sciences, University of Massachusetts Amherst, Amherst, MA USA; 7 Department of Health Promotion and Policy, School of Public Health and Health Sciences, University of Massachusetts Amherst, Amherst, MA USA

**Keywords:** Diseases, Endocrinology, Genetics, Medical research, Risk factors

## Abstract

The causal role of established risk factors for premenstrual disorders (PMDs) remains unclear. We used Mendelian randomization (MR) to assess causality for eight known-risk factors identified through a literature review. Summary statistics for these risk factors were from genome-wide association studies (GWAS) with sample size ranging from 129,017 to 1.2 million, and for PMDs from a GWAS of 72,297 participants. Findings were validated using one-sample MR in LifeGene cohort (*n* = 5674–5937). In two-sample MR, genetic liability to smoking initiation (OR = 1.25 (1.10–1.43)), earlier menarche (OR = 0.94 (0.89–0.98) per year), and higher BMI (OR = 1.19 (1.05–1.34) per kg/m^2^) were associated with PMD risk. No causal association was indicated for anemia, childhood abuse, childhood asthma, diabetes, and endometriosis. In one-sample MR, point estimates for BMI (OR = 1.10 (0.88–1.38) per kg/m²) and earlier menarche (OR = 0.98 (0.83–1.15) per year) were directionally consistent with the two-sample MR findings, but the confidence intervals included null effects. However, a null association was observed for smoking (OR = 0.94 (0.77–1.16)). The two-sample MR supports causal role of earlier menarche, higher BMI, and smoking in risk of PMDs. The lack of replication in one-sample MR highlights the need for triangulating evidence from future well-powered and methodologically comparable studies to strengthen these findings.

## Introduction

Premenstrual disorders (PMDs) are characterized by emotional and somatic symptoms before menses. PMDs can be classified into premenstrual syndrome (PMS), which affects 20–30% of women, and the more disabling form, premenstrual dysphoric disorder (PMDD), with an estimated prevalence of 2–6%^1,2^. Although PMDs only occur during reproductive ages, studies have shown they are associated with long-term health consequences, including early menopause, severe menopause symptoms^3^, cardiovascular diseases^4^, and suicide^5^.

Knowledge on risk factors of PMDs can provide insights into disease mechanism and inform prevention or intervention strategies. A handful of risk factors have been identified for PMDs. For example, lifestyle factors (e.g., cigarette smoking^6^, obesity^7,8^, nutrient intake^9–11^), diseases (e.g., childhood asthma^12^), reproductive traits (e.g., early puberty^13^), and psychosocial factors (e.g., adverse childhood experiences^14^ and psychological stress^15^) have been associated with risk of PMDs. However, current evidence is mainly based on observational studies that may be limited by reverse causation and residual confounding, and therefore less informative on the causal nature of these associations.

Using genetic variants as instrumental variables for exposure can minimize bias from reverse causation and residual confounding, and thus make it possible to estimate causal association between exposures and outcomes^16–20^. However, this method, known as Mendelian randomization (MR)^21^, has not been employed to investigate causal risk factors for PMDs. Here, to assess causality for known-risk factors of PMDs, we first performed a two-sample MR on risk factors identified from a literature review, and then validated the results in one-sample MR in a large Swedish cohort.

## Methods

We reported the study following Strengthening the Reporting of Observational Studies in Epidemiology using Mendelian Randomization (STROBE-MR)^22^. The study is approved by the Swedish Ethical Review Authority (2025-02016-01).

### Literature review on known risk factors

Our previous literature review has summarized risk factors of PMDs identified by prospective studies (Supplementary Table 1)^23^. After excluding exposures with no genome-wide association study (GWAS) available, we included 8 exposures/risk factors in the present analyses, including anemia, age at menarche (AAM), BMI, childhood asthma, childhood abuse, diabetes, endometriosis, and cigarette smoking.

### Two-sample Mendelian randomization

We searched the latest and largest GWAS for each exposure among European-ancestry individuals (Supplementary Table 2). Studies which provided female-specific results were preferred; in our study, we used female-only GWAS for AAM, BMI, diabetes and endometriosis. There is minimal sample overlap between exposure and outcome GWAS population. After extracting genetic variants associated with the exposure at genome-wide significance threshold (*p* < 5 × 10⁻⁸), we removed palindromic SNPs and SNPs in linkage disequilibrium (R^2^ ≥ 0.01 within 10,000 kb window)^24,25^. Given evidence of pleiotropy in the genetic instrument for BMI, in an additional analysis, we excluded SNPs associated with traits other than BMI at genome-wide significance based on the GWAS Catalog.

In our previous study^26^, we performed a GWAS of probable PMDs among 17,511 cases and 54,786 controls from European-ancestry women. The analysis was based on the LifeGene cohort in Sweden^27^ (*N* = 5229), and Mother, Father and Child Cohort Study (MoBa) cohort in Norway (*N* = 67,068)^28,29^, and the results were predominated driven by MOBA. PMDs were measured using questionnaires and register-based clinical diagnoses. One SNP was identified as genome-wide statistically significant and the SNP-based heritability was 7.2%.

We harmonized genetic instruments and ensured that the effect estimate corresponds to the same allele. To estimate causal associations, we used the random-effect inverse-variance weighted (IVW) as the main analysis, with weighted median^30^, weighted mode^31^, MR-Egger^32^, and Mendelian Randomization Pleiotropy RESidual Sum and Outlier (MR-PRESSO)^33^ as complementary analyses. These methods have different assumptions on horizontal pleiotropy and consistent results across different methods reduces the likelihood of false-positive findings^34^. Statistical power was calculated based on https://sb452.shinyapps.io/power. We applied the Benjamini & Hochberg method to control the false-positive rate per method^35^. A two-sided adjusted *p* value < 0.05 was considered as statistically significant. Analyses were performed in R 4.4.2.

MR studies have 3 assumptions: (1) the genetic instrument is related to the exposure (relevance); (2) there is no confounding of the genetic instrument-outcome association (independence); (3) the genetic instrument is associated with the outcome only through the exposure (exclusion restriction)^36^. To test the assumption of relevance, we calculated R^2^ and F statistics. The assumption of independence should hold since genes are randomly allocated at birth^37^. In addition, we evaluated horizontal pleiotropy by assessing heterogeneity between SNPs using Cochran’s Q statistic, estimating intercept in MR egger regression^32^ and performing MR PRESSO global test^33^.

### One-sample Mendelian randomization in LifeGene

AAM, BMI and cigarette smoking were identified as potential causal risk factors of PMDs in two-sample MR analysis. However, since the results in two-sample MR are potentially limited by the relatively simple assessment of PMDs, we complemented the findings in one-sample MR in LifeGene, in which PMDs were measured with a well-characterized questionnaire. In addition, to explore potential heterogeneity in these associations, we also estimated association with subtypes of PMDs, for which GWAS is not available. Given the limited statistical power in one-sample MR, the results should be interpreted as assessing directional consistency and exploring potential subtype heterogeneity.

The results from two- and one-sample MR analyses were not meta-analyzed due to non-independence between datasets and differences in exposure and/or outcome definitions across the two settings.

LifeGene is a large prospective cohort study in Sweden^27^. Briefly, during 2009-2019, index persons aged between 18 and 45 years and their household members were enrolled and sent questionnaires assessing lifestyles and health history. About half of the participants took physical examination and provided blood samples at a test center. We linked participants to Swedish registers through personal identification number. Informed consent was obtained at online registration or a test center.

In this analysis, we included female participants aged between 16 and 60 at enrollment, and who had menstruated within the last year, leaving 17,779 eligible women. We further restricted to unrelated individuals of European ancestry with available exposure and genotype data, resulting in 5674 women included the analysis for AAM, 5794 for BMI, and 5937 for smoking in the one-sample MR analysis.

At recruitment, participants recalled their AAM and reported if they had ever smoked more than 100 cigarettes. BMI was calculated based on self-reported height and weight. For a subset of women who had ever been pregnant (*n* = 94), BMI information was obtained from the Medical Birth Register^38^ if data were missing in the survey.

Information on genotyping and quality control in LifeGene is described elsewhere^26^. Briefly, we applied standard GWAS quality control by including SNPs which had minor allele frequency (MAF) ≥ 1%, genotyping rate ≥ 99%, *p* value from the Hardy-Weinberg Equilibrium ≥1e–6, and imputation quality score (INFO) ≥ 0.9^39^. We built a polygenic risk score (PRS) as an instrumental variable for exposures^39^, using 1000 Genomes Project data as reference data^40^. Specifically, we extracted non-ambiguous, independent (R^2^ = 0.01, distance threshold = 10,000 kb), and genome-wide significant variants (*p* < 5 × 10⁻⁸) from the most recent and largest GWAS^41–43^, i.e., the same GWAS used to derive the genetic instruments in two-sample MR analyses for the corresponding exposures. For BMI, SNPs associated with traits other than BMI at genome-wide significance were further excluded. Then, we multiplied the number of effect alleles at each SNP by effect size in the GWAS summary statistics, and then standardized it to a *z* score.

PMDs were assessed by a modified version of the Premenstrual Symptom Screening Tool (PSST)^44^. The original PSST has a high sensitivity (79%) but a low specificity (33%)^45^. The modified PSST in LifeGene had three screening questions, asking whether participants experienced symptoms that: (1) occur before menstruation, (2) affect relationships and daily activities, and (3) are absent after menstruation. Due to these screening questions, PMDs identified in LifeGene are mainly PMDD and severe PMS. Participants who endorsed all the screening questions were asked to indicate the severity and impact of 15 premenstrual symptoms. Consistent with previous studies^46^, we identified women with probable PMDs and then classified these further into severe PMS and PMDD (Supplementary Table 3).

To supplement PMD cases, we further identified clinical diagnoses of PMDs at baseline from the National Patient Register, Regional Primary Care Register, and National Prescribed Drug Register^4,47^, covering both specialist and primary care data in Sweden (Supplementary Table 4).

In MoBa and LifeGene, PMDs were identified based on questionnaire data and clinical diagnoses; however, due to the specific identification approaches differed, LifeGene likely captured a more refined and severe PMD phenotype whereas MoBa may include individuals with milder symptoms (Supplementary Table 5). Therefore, we believe findings from two- and one-sample were not fully comparable and considered one-sample analyses as complementary to assess directional consistency.

We collected information on age, income, civil status, country of birth, place of residence, education level, alcohol drinking, parity, physical activity, childhood abuse, use of oral contraceptives (OC), depression and anxiety diagnoses from LifeGene and/or registers (Supplementary Table 6). Since individuals included were genotyped by 4 different sub-studies, we also collected information on sub-study membership.

We first estimated the phenotypic association of AAM, BMI and smoking with PMDs using multivariable logistic regression.

We used a 2-step instrumental variable analysis to estimate causal associations^48^. First, we used logistic regression to estimate genetically predicted likelihood of smoking, and linear regression to estimate genetically predicted AAM and BMI, using their respective PRS as predictors. Then, we used logistic regression to estimate associations between genetically predicted exposures and PMDs. The genetically predicted probability of smoking was standardized to reduce variance^49,50^. Age, the first 10 principal components and substudy membership were adjusted in each stage of model.

To illustrate potential different causal associations for subtypes of PMDs, we estimated the association with (1) severe PMS and PMDD, and (2) PMDs comorbid with/without history of depression or anxiety.

To test the relevance assumption, we calculated OR/beta, R^2^ and F statistics for exposures. The independence assumption should hold since we have adjusted for population stratification. To further test pleiotropy of exposures on subtypes of PMDs, similar to previous studies^51,52^, we performed IVW, MR Egger, weighted mean and weighted mode analyses.

In addition, in one-sample MR in LifeGene, PMDs were identified by questionnaire and register-based clinical diagnoses; the latter may capture more severe PMDs. We therefore analyzed the association separately for PMDs identified by different approaches. Furthermore, for smoking, the point estimate in one-sample MR was directionally inconsistent with two-sample MR. To address the concern of sex mismatch, i.e., using sex-combined GWAS to build female-specific PRS as genetic instrument for smoking, we additionally performed analyses using female-specific GWAS of smoking initiation derived from UK Biobank^53^.

Analyses were performed using R 4.4.2, plink 1.9.0^54^, and SAS 9.4. We calculated the statistical power. Since the one-sample MR aimed to confirm rather than generate hypotheses, we did not adjust for multiple comparison. A two-sided *p* value < 0.05 was considered as statistically significant.

## Results

### Two-sample Mendelian randomization

**Fig. 1:**
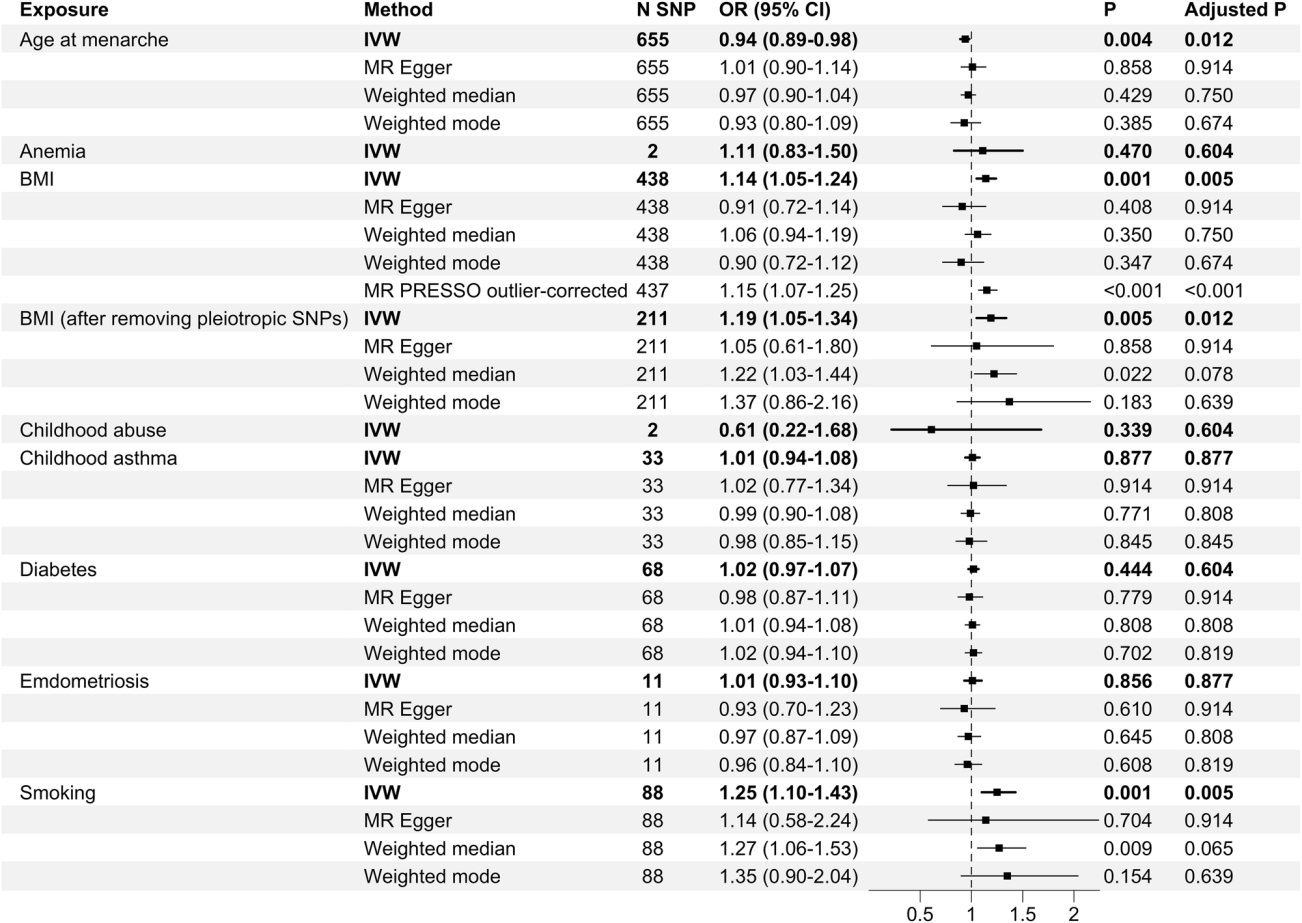
Causal associations between exposures and PMDs in two-sample MR. BMI body mass index, CI confidence interval, N SNPs number of single nucleotide polymorphisms, OR odds ratio. MR PRESSO outlier-corrected regression was only performed when there is outlier SNP identified. *P* values were adjusted based on Benjamini & Hochberg per method.

Genetic liability to smoking (OR_IVW_ = 1.25 (1.10–1.43) per unit, *p*_adjusted_ = 0.005) and earlier menarche (OR_IVW_ = 0.94 (0.89–0.98) per year, *p*_adjusted_ = 0.012) were associated with increased risk of PMDs (Fig. 1). For both exposures, the genetic instruments are strong (Supplementary Table 7) and the effect direction was generally consistent across different methods (Fig. 1). Although Cochran’s Q and MR-PRESSO global test suggested pleiotropy among SNPs (*p* < 0.05), the MR-Egger test did not indicate directional pleiotropy (*p* for intercept >0.05), suggesting the pleiotropic effects might be balanced across SNPs (Supplementary Tables 8–9).

Genetic liability to a higher BMI was associated with a higher risk of PMDs in IVW analyses (OR_IVW_ = 1.14 (1.05–1.24) per kg/m^2^, *p*_adjusted_ = 0.005), whereas the association was not consistent when using other methods (e.g., OR_MR-Egger_ = 0.91 (0.72–1.14), *p*_adjusted_ = 0.914) (Fig. 1). Heterogeneity was indicated in Cochran’s Q test (*p* < 0.05) and MR-Egger suggested a directional, albeit small, pleiotropic effect (intercept = 0.005 (0.000–0.009), *p* = 0.039) (Supplementary Tables 8–9). In addition, MR PRESSO identified 1 outlier SNP but the outlier-corrected association (OR = 1.15 (1.07–1.25)) was similar to IVW (*p* for distortion test = 0.815). However, after removing 227 out of 438 SNPs associated with additional traits (e.g., blood pressure, fatty acid level, etc; summarized in Supplementary Table 10), IVW analysis supported a positive association between genetic liability to a higher BMI and PMDs (OR_IVW_ = 1.19 (1.05–1.34) per kg/m^2^, *p*_adjusted_ = 0.012), and the effect direction was consistent across different methods. There was no evidence indicating heterogeneity (*p* > 0.05) or directional pleiotropy (MR Egger intercept = 0.002 (–0.007,0.011), *p* = 0.651).

No association with PMDs was indicated for genetic liability to anemia, childhood abuse, childhood asthma, diabetes, or endometriosis (Fig. 1).

### One-sample Mendelian randomization in LifeGene

Among 6003 participants, 1058 (17.6%) were identified as having a PMD, among which 894 (84.5%) were identified based on questionnaire only, 54 (5.1%) through register-based diagnosis only, and 110 (10.4%) through both approaches. The mean age at baseline among women with PMDs was 34.5 ± 8.1 years (Supplementary Table 11). When analyzing the phenotypic associations, AAM (OR = 0.93 (0.88–0.98)) and smoking (OR = 1.42 (1.23–1.64)) were associated with PMDs (Supplementary Fig. 1). However, a null association with PMDs was observed for BMI, assessed both continuously and categorically.

**Fig. 2:**
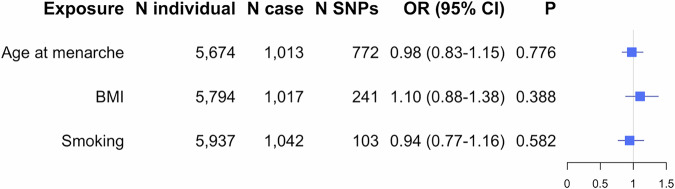
Causal associations of age at menarche, BMI, and smoking with PMDs in LifeGene. BMI body mass index, CI confidence interval, N SNPs number of single nucleotide polymorphisms, OR odds ratio. Analyses were performed on unrelated individuals with European ancestry who aged 16–60 at enrollment, had menstruated in the past year, and had genotype and exposure data. Severe PMS and PMDD were identified in LifeGene only. The ORs for smoking refer to OR per SD increase in standardized predicted probability of smoking, and ORs for AAM and BMI refer to OR per unit (year for AAM and kg/m^2^ for BMI) increase in their predicted values.The number of included SNPs is larger than Fig. 1 because in two-sample analysis, we followed the R pipeline where ambiguous SNPs were excluded after preforming LD clumping, whereas in one-sample analysis, we removed ambiguous SNPs before preforming LD clumping, to retain more SNPs and improve the predictive value of PRS.

Genetically predicted BMI (OR = 1.10 (0.88–1.38) per kg/m²) and AAM (OR = 0.98 (0.83–1.15) per year) showed directionally consistent point estimates as the two-sample MR analyses, whereas the confidence intervals were compatible with null effects (Fig. 2). In contrast to results in the two-sample MR (OR = 1.25 (1.10–1.43)), which corresponded to OR of 1.12 (1.05–1.20) per SD in smoking after scale conversion, a null association was found between genetically predicted liability to smoking and PMDs OR = 0.94 (0.77–1.16) per SD increase in standardized predicted probability of smoking (Fig. 2). However, the PRS of smoking explained 0.5% variance (Supplementary Table 12) and the statistical power was 7% (Supplementary Table 13). In addition, the results using female-specific GWAS showed directionally consistent results (Supplementary Table 14).

When analyzing by ascertainment source of PMDs, we observed a seemingly stronger point estimate for PMDs ascertained in register, compared to LifeGene, whereas the confidence interval overlapped (Supplementary Fig. 2).

**Fig. 3:**
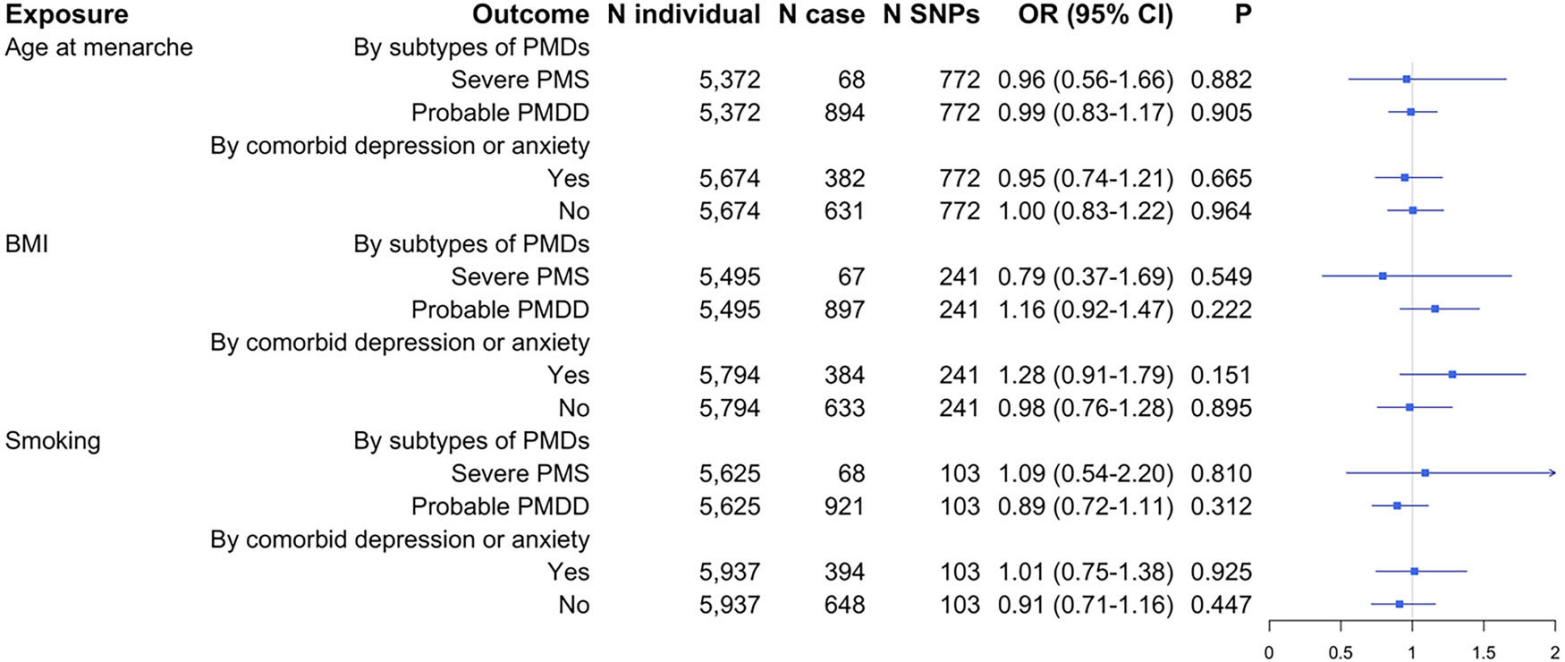
Causal associations of age at menarche, BMI, and smoking with subtypes of PMDs in LifeGene. BMI body mass index, CI confidence interval, N SNPs number of single nucleotide polymorphisms, OR odds ratio, PMS premenstrual syndrome, PMDD premenstrual dysphoric syndrome. Analyses were performed on unrelated individuals with European ancestry who aged 16–60 at enrollment, had menstruated in the past year, and had genotype and exposure data. Severe PMS and PMDD were identified in LifeGene only. The ORs for smoking refer to OR per SD increase in standardized predicted probability of smoking, and ORs for AAM and BMI refer to OR per unit (year for AAM and kg/m^2^ for BMI) increase in their predicted values.

When analyzing subtypes of PMDs, no statistically significant association was found for AAM, BMI, and smoking in subtype analyses. However, a positive association was indicated between BMI and PMDs with a history of depression or anxiety in phenotypic analyses (OR = 1.04 (1.00–1.08), *p* = 0.045; Supplementary Fig. 1). In one-sample MR analyses, a positive point estimate was indicated, but the confidence interval was compatible with no effect (OR = 1.28 (0.91–1.79); Fig. 3). However, the association between genetically predicted BMI and PMDs with a history of depression or anxiety was not consistent in other methods (Supplementary Table 15).

## Discussion

Our study, based on two-sample MR, found evidence indicating potential causal associations of AAM and BMI with PMDs, and the point estimates from the one-sample MR for BMI and AAM were directionally consistent with the two-sample MR findings, although the confidence intervals were compatible with null effects. Moreover, the causal association between smoking initiation and PMDs was indicated in two-sample MR, yet the association attenuates in one-sample MR. No causal associations were found for anemia, childhood abuse, childhood asthma, diabetes, and endometriosis.

Previous studies reported conflicting results on phenotypic association between AAM and PMDs. While one reported a positive association^55^, two suggested a U-shaped association^1,56^, and others reported an inverse association^13,57,58^. In our study, we found an inverse association between AAM and PMDs in both phenotypic analyses (OR = 0.93 (0.88–0.98)) and two-sample MR analyses (OR_IVW_ = 0.94 (0.89–0.98)). The direction of the association was consistent in one-sample MR in LifeGene, although the confidence interval included null effect, likely because of low statistical power.

There are some biological explanations to the potential causal association between menarche timing and PMDs. First, the activation of gamma-aminobutyric acid subtype A (GABA_A_) receptors, located on gonadotropin-releasing hormone neurons, may be important to puberty onset^59^. In addition, the subunit composition of GABA_A_ receptor in response to luteal hormone changs is considered as a key mechanism underlying PMDs^60^. Second, earlier age at menarche is associated with a heighted inflammatory profile^61^, which has been observed in women with PMDs as well^62^. Indeed, inflammation may reduce level of allopregnanolone (ALLO) by shifting progesterone metabolism towards corticosterone synthesis and away from ALLO production^63^, thereby accelerating the decline in ALLO and potentially be related to abnormal response to hormone withdrawal in the late luteal phase.

Several observational studies reported a positive association between adiposity and PMDs^7,64^. In our study, the phenotypic association between BMI and PMDs was 1.01 (0.99–1.04), whereas a positive association was found for genetic liability to BMI and risk of PMDs in two-sample MR. Although pleiotropy was indicated in the initial analysis, the association remained after removing pleiotropic SNPs, supporting that a higher BMI has a potential causal effect on PMDs. A directionally consistent point estimate was indicated in one-sample MR in LifeGene, lending some further support to the potential causal association between BMI and PMDs.

Higher BMI can be associated with PMDs through several mechanisms. First, several studies found an inverse association between BMI and estradiol levels^65,66^. Estrogen can promote serotonin function by enhancing its synthesis, transport and responsiveness. It is likely that lower level of estradiol related to adiposity may impair serotonin function, and lead to the development of PMDs^67^. In addition, adiposity may affect risk of PMDs through increased inflammation^62,68^, and dysregulated renin-angiotensin-aldosterone system^69,70^. Since BMI is a modifiable factor, future studies may be warranted to study if weight loss reduce risk of PMDs.

Notably, in LifeGene, the point estimate between genetically predicted BMI and PMDs with a history of depression and anxiety was directionally positive, although the confidence interval was compatible with no effect. However, the inconsistent association across different methods indicates potential pleiotropy. PMDs with a history of depression and anxiety may represent premenstrual exacerbation (PME) of underlying psychiatric conditions^71^. Future studies are needed to elucidate the causal pathways linking BMI, psychiatric disorders and PMDs.

A meta-analysis estimated a pooled OR of 1.56 (1.25–1.93) for the association between smoking and PMDs^72^, which is similar to the phenotypic association in our study (OR = 1.42 (1.23–1.64)). However, in our study, the two-sample MR suggests an OR of 1.25 (1.10–1.43) in IVW analysis. Such difference may be related to residual confounding which cannot be fully controlled in observational studies. However, the causal estimate in our study may not be directly compared to those derived from observational studies. First, smoking is a binary exposure that may represent an underlying continuous liability (e.g., the likelihood to smoke)^73^. Second, genetic instruments reflect lifelong differences in smoking, whereas conventional observational analyses may reflect smoking at a specific time point.

Notably, we did not replicate the findings for smoking in one-sample MR. Although the smoking PRS explained 0.5% variance in smoking, the instrument strength was acceptable (F statistic = 32), suggesting that weak instrument bias alone is unlikely to explain the discrepant findings between the one- and two-sample MR analyses. Several other factors may also contribute to these differences. First, although classic winner’s curse is less likely because SNPs were selected from an external GWAS and the PRS-smoking association was independently estimated in LifeGene, inflated SNP effect estimates in the discovery GWAS may still have weakened instrument strength in the one-sample MR analysis. Second, the smoking phenotypes differed across the two settings. Specifically, while the external GWAS captured broader smoking initiation and regular smoking behavior^43^, LifeGene defined smoking as ever smoking more than 100 cigarettes. Third, the one-sample MR analysis was substantially underpowered compared with the two-sample MR analysis, resulting in wider confidence intervals and lower precision. Finally, the genetic instruments were derived from a sex-combined international consortium, whereas the one-sample MR analysis was restricted to Swedish women in LifeGene, who may have different smoking behaviors (e.g., greater snus use). Indeed, when we performed sensitivity analyses using a female-specific GWAS of smoking initiation, the point estimate in one-sample was directionally consistent with the two-sample MR findings, emphasizing the importance of using sex-matched GWAS in one-sample MR analyses. Future studies with improved statistical power and greater comparability in population characteristics and smoking definitions, especially using sex-matched instruments, are needed to confirm these associations.

Despite this, there can be several biological pathways to explain the potential causal association as indicated in the two-sample MR. Smoking induced decreased stress response of hypothalamic-pituitary-adrenal (HPA) axis^74^. A dampened cortisol response was also found among women with PMDs irrespective of menstrual phases^75,76^. Furthermore, smoking is associated with chronic inflammation^77^, which is involved in etiology of PMDs. In addition, smoking has effects on levels of female steroid hormone^78^, but future studies are warranted to study how this may affect downstream hormone sensitivity.

We did not find a causal association for anemia, childhood abuse, childhood asthma, diabetes and endometriosis with PMDs. It is likely previous studies are biased by residual confounding. However, for anemia, no SNP was included after harmonizing data from GWAS for anemia^79^ and PMDs; therefore, we used GWAS for iron deficiency anemia (IDA) instead^80^. As a result, our findings reflect the causal association for IDA with PMDs, and may not be comparable to the phenotypic association. In addition, the GWAS of childhood abuse were performed on a relatively small population^81^, which may result in fewer numbers of genetic instruments and power imbalance between exposures. Future studies using larger GWAS studies are warranted to validate the null results.

Built on the first GWAS study of PMDs, our study is the first to use a genetically informed approach to comprehensively assess causal risk factors of PMDs. By triangulating MR findings from both summary-level and individual-level data, we were able to cross-validate results and strengthen the robustness of our findings.

In addition to the limited statistical power in one-sample MR mentioned above, our study has other limitations. First, many exposures in our study are binary, which are dichotomization of latent continuous variables. This puts limits on the exclusion-restriction assumption, since there can be an alternative pathway from genetic instrument to outcome through the underlying continuous variable^73^. Although approaches such as multivariable MR may help address pleiotropic pathways, they may weaken the effect of the categorical exposure as well. However, our tests of causal association should still be valid^73^. Second, instead of prospective charting, PMDs were identified using retrospective questions and clinical diagnoses. However, although the validity of PMDs was unknown, for some exposures, the results in the one-sample MR in LifeGene - where most of PMDs are identified based on a well-characterized questionnaire^47^- are consistent with two-sample MR, in which the GWAS of PMDs was predominated by MoBa, where PMDs were measured simply by two questions^82^. Such consistency supported the robustness of our results. Third, in the GWAS of PMDs, the SNP-based heritability was 7.2%, which was lower than the heritability of PMDs reported in twin studies (35–56%)^83–86^. Although misclassification of PMDs should be considered due to invalidated assessment^83–85^, the missing heritability may indicate many relevant SNPs remained undetected and potentially biased the association towards null in two-sample MR. Fourth, LifeGene is different from general population in Sweden in terms of demographics and smoking^87^. If participation is also related to probability of having a PMDs, selection bias would be introduced. However, the phenotypic association in our study was similar to other studies, indicating selection bias, if any, should not severely distort our results. Fifth, in the analyses of BMI, we applied a strict filtering strategy to exclude SNPs associated with additional anthropometric, cardiometabolic, inflammatory, or metabolic traits because of concerns regarding horizontal pleiotropy. However, some excluded variants may also reflect biologically relevant pathways linking BMI to PMDs, potentially resulting in conservative estimates. Last, our analyses were restricted to individuals of European ancestry, thus our findings may not generalize to other ancestries. Future studies incorporating diverse ancestry backgrounds are needed.

In conclusion, based on two-sample MR, we found evidence supporting potential causal effects of earlier menarche, adiposity, and smoking initiation on increased risk of PMDs. Despite this, the lack of replication in one-sample MR may reflect methodological limitations, and emphasizes the importance of triangulating well-powered and methodologically comparable evidence to enhance causal inference. If confirmed in future studies, it indicates incorporating information on pubertal timing, adiposity and smoking status may inform targeted prevention of PMDs.

## Supplementary information

Supplementary Information.

## Data Availability

Publicly available GWAS summary statistics were used for the two-sample MR analyses (see Supplementary Table S2).

## Code availability

The analyses codes are available from the corresponding authors upon request.
