# Supplementary material for: Mendelian randomization of risk factors for premenstrual disorders
Source: Npj Ment Health Res. 2026 Jul 16;5:35. doi: 10.1038/s44184-026-00231-4 (PMC13376395; doi:10.1038/s44184-026-00231-4)
Supplement: Supplementary file 1 — Supplementary Information. [file 44184_2026_231_MOESM1_ESM.pdf]

Table of Contents

Supplementary Table 1 Risk factors of PMDs identified from prospective studies 2

Supplementary Table 2 Description of GWAS used for each exposure 3

Supplementary Table 3 Assessment criteria for PMDs and PMDD in LifeGene 5

Supplementary Table 4 Ascertainment of PMDs in registers 6

Supplementary Table 5 Comparison of PMD definitions in MoBa and LifeGene 7

Supplementary Table 6 Ascertainment of covariates 8

Supplementary Table 7 Instrument strength in two-sample MR 9

Supplementary Table 8 Tests of heterogeneity for genetic instruments in two-sample MR 10

Supplementary Table 9 Tests of directional pleiotropic effects of the genetic instruments on PMDs in two-sample MR 11

Supplementary Table 10 SNPs removed in the analysis of BMI in two-sample MR 12

Supplementary Table 11 Baseline characteristics between individuals with and without PMDs, among individuals included in one-sample MR in LifeGene 19

Supplementary Table 12 Assessment of instrument strength for smoking and age at menarche in one-sample MR 21

Supplementary Table 13 Power calculation 22

Supplementary table 14 Sensitivity analysis using female-specific smoking GWAS 23

Supplementary Table 15 IVW, MR Egger, Weighted median and weighted mode regression for subtypes of PMDs 24

Supplementary Figure 1 Phenotypic associations of age at menarche, BMI and smoking with PMDs and its subtypes, among individuals included in one-sample MR in LifeGene ..26

Supplementary Figure 2 Causal associations of age at menarche, BMI and smoking with PMDs in one-sample MR in LifeGene, by different ascertainment approach of PMDs 27

Reference 28

Supplementary Table 1 Risk factors of PMDs identified from prospective studies

	Risk factors	Source study	Included in the present study
Nutrition intake	calcium	Bertone-Johnson, 2005	No[a]
	vitamin D		
	nonheme iron	Chocano-Bedoya, 2013	
	potassium		
	caffeine	Purdue-Smithe, 2016	
	carbohydrate	Houghton, 2018	
	fiber		
	protein	Houghton, 2019	
	total fat	Houghton, 2021	
Disease	anemia	Lee, 2022	Yes
	diabetes	Huang, 2022	Yes
	endometriosis	Gete, 2023	Yes
	childhood asthma	Yang, 2023	Yes
	childhood food allergies		No[b]
Women's health	age at menarche	Lu, 2021	Yes
	early pubic hair growth		No[b]
Others	BMI	Lu, 2022; Bertone-Johnson, 2010	Yes
	smoking	Bertone-Johnson, 2008	Yes
	childhood abuse[c]	Bertone-Johnson, 2014	Yes
	psychological stress	Martins, 2024	No[d]

[a] Most GWAS studies focused on serum levels of these nutritions and no derived phenotype is available in LifeGene.
[b] No GWAS available.
[c] In this study, the main analysis is not based on a prospective design, but a sensitivity analysis using a prospective design, by restricting to individuals who first reported PMDs after the exposure survey, generated similar results.
[d] No GWAS available.

Supplementary Table 2 Description of GWAS used for each exposure

Risk factors	GWA study	Consortium	Overlap with LifeGene and MoBa	Total sample size	Sex	Phenotype assessment	Covariates adjustment
Anemia	Toivonen, 2023[a,1]	FinnGen, and UK Biobank	No known overlap recognized	665,460	Male and female	ICD code	Study-specific
Age at menarche	Kentistou, 2024[2]	ReproGen, UK Biobank, the Breast Cancer Association Consortium and the Ovarian Cancer Association Consortium 23andMe	Some Nordic cohorts (e.g., Swedish Twin Registry) may have contributed to the consortium; however, substantial overlap with LifeGene or MoBa is unlikely	632,955	Female	Self-reports	Age and study-specific variables, e.g., genetic principal components
BMI	Pulit, 2019[3]	UK Biobank and GIANT	Some Nordic cohorts (e.g., Swedish Twin Registry, HUNT) may have contributed to the consortium; however, substantial overlap with LifeGene or MoBa is unlikely	434,794	Female	Calculated using height and weight	Study-specific
Childhood asthma	Sakaue, 2021[4]	UK Biobank and FinnGen	No overlap recognized	438,843	Male and female	ICD code	Study specific
Childhood abuse	Chen, 2025[b, 5]	UK Biobank	No overlap recognized	129,017	Male and female	Self-reported childhood maltreatment, inclding emotional abuse, emotional neglect, physical abuse, physical neglect, and sexual abuse	Genotyping batch and the top 20 principle components
Diabetes	Mahajan, 2018(b)[6]	DIAGRAM	Some Nordic cohorts may have contributed to the consortium; however, substantial overlap with LifeGene or MoBa is unlikely	464,389	Female	Vary by study e.g., self-reports, clinical diagnose and biochemical measurement	Population structure and relateness and study-specific variable
Endometriosis	Gualdo, 2025[7]	EstBB and FinnGen	No overlap recognized	293,618	Female	ICD code	Study specific

Smoking	Liu, 2019[8]	GSCAN	Some Nordic cohorts (e.g., HUNT) may have contributed to the consortium; however, substantial overlap with LifeGene or MoBa is unlikely	1,232,091	Male and female	The phenotype was asked in 3 ways: 1) Have you smoked over 100 cigarettes over the life? 2) Have you smoked each day for at least a month? 3) Have you smoked regularly?	In each study, age, age squared, sex, and genetic principal components were adjusted.

BMI, body mass index; DIAGRAM, DIAbetes Genetics Replication And Meta-analysis; EstBB, Estonian Biobank; GIANT, Genetic Investigation of Anthropometric Traits consortium; GSCAN, GWAS & Sequencing Consortium of Alcohol and Nicotine use.

[a]This study performed GWAS of iron deficiency anaemia.

[b]In this study, results on significant SNP among females for childhood abuse were provided, but since no SNP was included after applying the clumping strategy, we still used the sex-combined results for childhood abuse.

Supplementary Table 3 Assessment criteria for PMDs and PMDD in LifeGene

Assessment criteria of PMDs in LifeGene	Assessment criteria of PMDD in LifeGene
1) had at least one affective symptoms rated as moderate or severe;<br>2) had at least four additional symptoms rated as moderate or severe; and<br>3) had at least one symptom with moderate or severe influence on relationships or social life.	1) had at least one affective symptoms rated as severe;<br>2) had at least one symptom severely influencing relationships or social life.

Supplementary Table 4 Ascertainment of PMDs in registers

Data source	Code
National Patient Register and primary care register in Stockholm (>80% participants lived in Stockholm county)	ICD codes N943
Prescribed Drug Register	ATC codes of N06AB, N06AX, N06AA, G02B, G03A, with key words including "PMS", "PREMENSTRUELLT SYNDROM", "PREMENSTRUELLT DYSFORSIKT SYNDROM", "PREMENSTRUELLT DYSFORI", "PMD", "PMDD", "PMDS", "MENS" (for antidepressants only).

Supplementary Table 5 Comparison of PMD definitions in MoBa and LifeGene

	MoBa	LifeGene
Questionnaire-based assessment		
Questionnaire items	At the 15th week of gestation, women were asked if they felt depressed or irritable before menses; if so, whether such symptoms disappear after menses	Participants were asked if they had symptoms that occur before menstruation, affect daily activities, and are absent after menstruation. Upon confirmation, they were asked to indicate the severity and impact of 15 premenstrual symptoms.
criteria	Participants who confirmed both questions.	(1) had at least one affective symptoms rated as moderate or severe; (2) had at least four additional symptoms rated as moderate or severe; and (3) had at least one symptom with moderate or severe influence on relationships or social life.
Register-based assessment		
Data sources	Primary Care Registry of Norway	Swedish National Patient Register (NPR), primary care register in Stockholm, and Swedish Prescribed Drug Register
Codes	X89	In NPR and primary care register: N943;<br>In Prescribed Drug Register: searched ATC codes of N06AB, N06AX, N06AA, G02B, G03A, with key words including "PMS", "PREMENSTRUELLT SYNDROM", "PREMENSTRUELLT DYSFORSIKT SYNDROM", "PREMENSTRUELLT DYSFORI", "PMD", "PMDD", "PMDS", "MENS" (for antidepressants only).

Supplementary Table 6 Ascertainment of covariates

Variable	Ascertainment approach
birth year	derived from Total Population Register (TPR) and LifeGene questionnaire
household disposable income, civil status and educational level at the year before enrollment	derived from longitudinal integrated database for health insurance and labour market studies (LISA)
civil status	Derived from LifeGene questionnaire
country of birth	Derived from TPR
place of residence	Derived from TPR
physical activity	Derived by multiplying the time spent in each sport by its metabolic equivalent of task (MET) score, and then summed these values and generated a total MET score for each individual.
Parity	Medical Birth Register (MBR)
Alcohol intake, experience of childhood abuse, and use of oral contraceptives	collected at enrollment in LifeGene questionnaires
Depression	Derived using ICD code F32-F29 from National Patient Register (NPR) and primary care register in Stockholm, as well as using ATC code N06A from Prescribed Drug Register (PDR).
Anxiety	Derived using ICD code F40-F41 from NPR and primary care register in Stockholm, as well as prescription of anxiolytics (ATC code: N05B) in PDR.

Supplementary Table 7 Instrument strength in two-sample MR

Exposure	R^2 (%)	F statistics
Age at menarche	17,8	181
Anemia	0,5	1572,3
BMI	6,1	57,7
BMI (after remove pleiotropic SNPs)	2,3	43
Childhood abuse	0,1	33,4
Childhood asthma	8,4	1219,8
Diabetes	21,1	379,2
Endometriosis	4,7	1322,3
Smoking	2,3	168,2

F staitiscs was calculated by the formula $\boldsymbol{F} = \frac{(\boldsymbol{n}-\boldsymbol{k}-\mathbf{1})}{\boldsymbol{k}}\frac{\boldsymbol{R2}}{(\mathbf{1}-\boldsymbol{R2})}$, where R2 is the variance in the exposure explanied by genetic instruments, k is the number of instruments, and n is the average of effective sample size for each SNP (for childhood asthma, childhood abuse, endometriosis and anemia, sample size for each SNP was not available so we used total sample size instead).

Supplementary Table 8 Tests of heterogeneity for genetic instruments in two-sample MR

Exposure	Method	Q	df	P value
Age at menarche	MR Egger	755,9	653	0.003
Age at menarche	Inverse variance weighted	758,1	654	0.003
Anemia	Inverse variance weighted	2	1	0.159
BMI	MR Egger	526,3	436	0.002
BMI	Inverse variance weighted	531,4	437	0.001
BMI (after removing pleiotropic SNPs)	MR Egger	224,1	209	0.225
BMI (after removing pleiotropic SNPs)	Inverse variance weighted	224,4	210	0.236
Childhood abuse	Inverse variance weighted	1	1	0.317
Childhood asthma	MR Egger	36,1	31	0.243
Childhood asthma	Inverse variance weighted	36,1	32	0.283
Diabetes	MR Egger	98,5	66	0.006
Diabetes	Inverse variance weighted	99,1	67	0.007
Endometriosis	MR Egger	11,1	9	0.269
Endometriosis	Inverse variance weighted	11,6	10	0.316
Smoking	MR Egger	113,1	86	0.027
Smoking	Inverse variance weighted	113,2	87	0.031

BMI, body mass index.

Supplementary Table 9 Tests of directional pleiotropic effects of the genetic instruments on PMDs in two-sample MR

	MR Egger intercept test		MR PRESSO global test	
Exposure	Intercept	P value	Residual	P value
Age at menarche	-0.002 (-0.006,0.001)	0.171	760,5	0.003
Childhood abuse	/	/	/	/
Anemia	/	/	/	/
Childhood asthma	-0.001 (-0.022,0.020)	0.943	37,9	0.297
BMI	0.005 (0.000,0.009)	0.039	534	0.002
BMI (after removing pleiotropic effects)	0.002 (-0.007,0.011)	0.651	226,4	0.246
Diabetes	0.004 (-0.008,0.015)	0.534	101,8	0.008
Endometriosis	0.009 (-0.021,0.039)	0.555	13,7	0.355
Smoking	0.002 (-0.015,0.020)	0.784	116	0.036

BMI, body mass index.

Supplementary Table 10 SNPs removed in the analysis of BMI in two-sample MR

SNP	Associated traits
rs1003081	Lipid/fatty acid
rs10048652	Metabolic/endocrine dysfunction
rs10118701	hip circumference
rs1016287	Cardiometabolic traits
rs10182181	Lifestyle/behavioral traits; Metabolic/endocrine dysfunction
rs10237317	body surface area
rs10271582	Lifestyle/behavioral traits
rs1038088	body fat percentage; Lipid/fatty acid
rs10448285	facial morphology trait; Musculoskeletal traits; open-angle glaucoma
rs1048932	Metabolic/endocrine dysfunction; Musculoskeletal traits; overnutrition, obesity
rs10499694	Metabolic/endocrine dysfunction
rs10513801	Inflammation/hematologic traits; Lipid/fatty acid; serum creatinine amount, glomerular filtration rate; waist circumference
rs10756714	visceral adipose tissue quantity
rs10761785	anthropometric, cardiovascular, inflammatory, lipid-related, and circulating protein traits
rs10875633	Neurological/psychiatric dysfunction
rs10920678	Metabolic/endocrine dysfunction; wellbeing measurement
rs10930502	body fat percentage; fat pad mass; Lifestyle/behavioral traits; Metabolic/endocrine dysfunction
rs10938397	adiposity, body composition, reproductive, cardiometabolic, lipid-related, and metabolic traits.
rs10962552	behaviour
rs11066188	cardiovascular and coagulation-related traits
rs11074446	Metabolic/endocrine dysfunction
rs11165643	body fat percentage; Lifestyle/behavioral traits; Lipid/fatty acid; Metabolic/endocrine dysfunction
rs11218510	body height
rs112566467	body fat percentage; fat pad mass
rs1160983	Lipid/fatty acid; Neurological/psychiatric dysfunction; sexual dimorphism measurement
rs11611246	Metabolic/endocrine dysfunction
rs11660335	Metabolic/endocrine dysfunction
rs11672660	metabolic, renal, anthropometric, and lifestyle-related traits
rs11677607	Metabolic/endocrine dysfunction
rs1167800	mathematical ability; Metabolic/endocrine dysfunction
rs11692326	heart failure; Metabolic/endocrine dysfunction
rs11751591	Lipid/fatty acid; multisite chronic pain; Pain
rs11824092	Lifestyle/behavioral traits
rs11855853	Educational/socioeconomic status; Metabolic/endocrine dysfunction
rs12042959	Metabolic/endocrine dysfunction; waist-hip ratio
rs12089815	Educational/socioeconomic status; frailty measurement; Lifestyle/behavioral traits; Neurological/psychiatric dysfunction

rs12140153	adiposity, body composition, metabolic, inflammatory, liver-related, and lifestyle traits.
rs12222235	Metabolic/endocrine dysfunction
rs12364470	Metabolic/endocrine dysfunction
rs12376870	body fat percentage; body height
rs12429545	Adiposity; Lifestyle/behavioral traits; Metabolic/endocrine dysfunction; waist circumference
rs12446632	puberty; adiposity; Lifestyle/behavioral traits; Lipid/fatty acid; Metabolic/endocrine dysfunction; Musculoskeletal traits
rs12462975	body height; calcium
rs12468070	Metabolic/endocrine dysfunction
rs12484438	body height; lean body mass; uterine fibroid
rs12514473	Metabolic/endocrine dysfunction
rs12530737	fat pad mass
rs12593036	Metabolic/endocrine dysfunction
rs12622013	Metabolic/endocrine dysfunction
rs12680842	Metabolic/endocrine dysfunction
rs12681792	body height; Metabolic/endocrine dysfunction
rs12692596	Reproductive trait; adiposity; Educational/socioeconomic status; Lipid/fatty acid; Metabolic/endocrine dysfunction
rs12788343	body height
rs12914489	Educational/socioeconomic status; Lifestyle/behavioral traits; Metabolic/endocrine dysfunction
rs1296328	adiposity; frailty measurement; Lifestyle/behavioral traits; Lipid/fatty acid; Metabolic/endocrine dysfunction; overnutrition
rs12981256	Metabolic/endocrine dysfunction
rs13062093	Adiposity; behavior; Metabolic/endocrine dysfunction
rs13107325	adiposity and anthropometric measures, blood pressure and cardiovascular biomarkers, renal and metabolic traits, inflammatory/immune markers, lung function, musculoskeletal and pain-related traits, neurological/psychiatric traits, and numerous circulating protein and metabolite levels.
rs13110266	Metabolic/endocrine dysfunction
rs13174863	body fat percentage; body height; fat pad mass
rs13191362	Lifestyle/behavioral traits; Lipid/fatty acid; Metabolic/endocrine dysfunction
rs1329733	Musculoskeletal traits
rs13389219	adiposity, cardiometabolic, lipid-related, endocrine, inflammatory, and diabetic complication traits
rs1362910	mathematical ability
rs141729694	Educational/socioeconomic status; adiposity; Neurological/psychiatric dysfunction
rs1417665	Metabolic/endocrine dysfunction
rs1436348	body fat percentage; body height; Lipid/fatty acid
rs1441264	body fat percentage; fat pad mass; hip circumference; Lipid/fatty acid; adiposity
rs1452075	Educational/socioeconomic status; Metabolic/endocrine dysfunction; adiposity
rs1465406	Metabolic/endocrine dysfunction

rs1477199	Metabolic/endocrine dysfunction; Musculoskeletal traits; Neurological/psychiatric dysfunction
rs1524445	base metabolic rate measurement; adiposity
rs1559677	Metabolic/endocrine dysfunction
rs1631026	body height; whole body water mass
rs16851483	Lifestyle/behavioral traits; Metabolic/endocrine dysfunction
rs17014375	Metabolic/endocrine dysfunction
rs17066842	Metabolic/endocrine dysfunction; sexual dimorphism measurement
rs17309825	feeling miserable measurement; Metabolic/endocrine dysfunction
rs17391694	adiposity and body composition traits
rs17405819	Lifestyle/behavioral traits
rs17522122	cardiovascular, inflammatory, behavioral, psychiatric, metabolic, and socioeconomic traits
rs17583459	body height
rs17724992	body height; Lifestyle/behavioral traits; Metabolic/endocrine dysfunction
rs17751061	blood protein amount
rs1808579	comparative body size at age 10, self-reported; hip circumference
rs1808629	adiposity; Lipid/fatty acid
rs1852006	body height; comparative body size at age 10, self-reported; Metabolic/endocrine dysfunction
rs1861412	Lifestyle/behavioral traits
rs1884389	Metabolic/endocrine dysfunction
rs1884897	BMI-adjusted waist circumference; body height; Lifestyle/behavioral traits
rs1928295	Lifestyle/behavioral traits
rs1949197	Educational/socioeconomic status; fat pad mass
rs1964926	body height
rs1967772	body height
rs197374	Metabolic/endocrine dysfunction
rs1998710	Lifestyle/behavioral traits
rs2045293	Blood pressure
rs2074625	body height
rs210139	hematological measurement; Inflammation/hematologic traits; potassium measurement
rs2112347	adiposity, renal, lipid-related, endocrine, and lifestyle-related traits
rs215669	body fat percentage; Lipid/fatty acid; Metabolic/endocrine dysfunction
rs2161097	Neurological/psychiatric dysfunction; waist-hip ratio
rs2170382	Lifestyle/behavioral traits
rs2185027	Metabolic/endocrine dysfunction
rs2190788	Metabolic/endocrine dysfunction
rs2228213	Inflammation/hematologic traits; Metabolic/endocrine dysfunction
rs2274782	Inflammation/hematologic traits
rs2279574	Educational/socioeconomic status; Lifestyle/behavioral traits; mathematical ability; Neurological/psychiatric dysfunction
rs2289705	atrial fibrillation
rs2299383	Metabolic/endocrine dysfunction
rs2357760	waist-hip ratio

rs2479106	Metabolic/endocrine dysfunction; polycystic ovary syndrome
rs247975	Inflammation/hematologic traits; Lipid/fatty acid; Metabolic/endocrine dysfunction; Musculoskeletal traits
rs2479958	fat pad mass
rs249612	hip circumference
rs2504236	Inflammation/hematologic traits
rs2516740	body height
rs2568958	Neurological/psychiatric dysfunction
rs2579998	Metabolic/endocrine dysfunction
rs2605603	Metabolic/endocrine dysfunction
rs2616192	Lifestyle/behavioral traits; Metabolic/endocrine dysfunction
rs2721965	body fat percentage
rs2781668	smoking behavior, lipid-related traits, neurological/psychiatric traits, and amino acid metabolism markers
rs2814992	Lipid/fatty acid
rs2861685	body fat percentage
rs2968864	QT interval
rs2984618	Metabolic/endocrine dysfunction
rs3003578	body height
rs3130048	dental caries; adiposity
rs329120	Educational/socioeconomic status; adiposity; Inflammation/hematologic traits; Lifestyle/behavioral traits; Liver-related traits; Musculoskeletal traits; Neurological/psychiatric dysfunction; sex hormones
rs329651	body height
rs33429	risk-taking behaviour
rs337637	Educational/socioeconomic status; gdnf family receptor alpha-3 measurement; Inflammation/hematologic traits; Metabolic/endocrine dysfunction
rs340025	body fat percentage; Lipid/fatty acid; Liver-related traits; sex hormone-binding globulin measurement
rs34292685	body height
rs3803286	adiposity; immunology; Lipid/fatty acid
rs3806114	body height; Metabolic/endocrine dysfunction
rs3810291	adiposity, body composition, reproductive, cardiometabolic, endocrine, and lipid-related traits.
rs3814883	adiposity; Inflammation/hematologic traits; lean body mass; Lifestyle/behavioral traits; Lipid/fatty acid; Metabolic/endocrine dysfunction; Musculoskeletal traits; Neurological/psychiatric dysfunction; sexual dimorphism measurement
rs41310284	Adiposity and lung function
rs4148155	neurological, inflammatory, hematologic, renal, and anthropometric traits
rs4240673	Metabolic/endocrine dysfunction; Musculoskeletal traits
rs4307239	body height
rs4379706	Metabolic/endocrine dysfunction
rs4482463	adiposity; frailty measurement; Metabolic/endocrine dysfunction
rs4527444	body height
rs4606726	hip circumference; severe acute respiratory syndrome, COVID-19
rs4783718	Inflammation/hematologic traits

rs4790292	body fat percentage; body height; Lipid/fatty acid
rs4834272	Lifestyle/behavioral traits
rs4857857	C-C motif chemokine 2 level; hematological measurement
rs4858223	Musculoskeletal traits
rs4864201	atrial fibrillation; Metabolic/endocrine dysfunction
rs4880341	Metabolic/endocrine dysfunction
rs4889606	fat pad mass; hip circumference
rs4929923	age at menarche; Lifestyle/behavioral traits; Metabolic/endocrine dysfunction; visceral adipose tissue quantity
rs5396	Educational/socioeconomic status
rs543874	adiposity, behavioral, reproductive, cardiometabolic, inflammatory, and metabolic traits
rs563296	Lipid/fatty acid; Metabolic/endocrine dysfunction; Musculoskeletal traits
rs56803094	body fat percentage; body height
rs57800857	body fat percentage; fat pad mass; gait quality; Lipid/fatty acid; waist circumference
rs6010784	Metabolic/endocrine dysfunction
rs6050446	body height; Lifestyle/behavioral traits; protein measurement
rs613872	behavioral, psychiatric, inflammatory, neurological, and socioeconomic traits
rs61813324	body fat percentage; body height; fat pad mass; Lipid/fatty acid; waist circumference
rs62106258	adiposity and body composition traits, as well as smoking behavior, age at menarche, atrial fibrillation, inflammatory traits, and other cardiometabolic traits
rs6265	adiposity, cardiometabolic, behavioral, and psychiatric traits.
rs6446187	Educational/socioeconomic status; Musculoskeletal traits; Neurological/psychiatric dysfunction; Pain
rs6477694	Metabolic/endocrine dysfunction
rs6493498	Inflammation/hematologic traits; Metabolic/endocrine dysfunction
rs6548237	cigarettes per day measurement
rs6567160	adiposity, body composition, cardiometabolic, endocrine, lipid-related, renal, and lifestyle-related traits
rs6720868	Metabolic/endocrine dysfunction
rs6725931	Metabolic/endocrine dysfunction
rs6829208	Metabolic/endocrine dysfunction
rs6857	adiposity, cardiometabolic, lipid-related, neurodegenerative, and neurological/psychiatric traits
rs6864049	Educational/socioeconomic status; Lifestyle/behavioral traits; Metabolic/endocrine dysfunction
rs6870983	body height; Inflammation/hematologic traits; Lifestyle/behavioral traits; mathematical ability; Metabolic/endocrine dysfunction; visceral adipose tissue quantity
rs6879711	Inflammation/hematologic traits
rs6890310	Metabolic/endocrine dysfunction
rs6964833	age at menarche
rs6973700	post-traumatic stress disorder
rs7024334	Lifestyle/behavioral traits; Metabolic/endocrine dysfunction
rs7084454	Carcinoma; Lipid/fatty acid; overnutrition, obesity

rs709400	Urate/renal traits
rs7105462	behavioral and gastrointestinal-related traits
rs7107409	Musculoskeletal traits
rs7124681	body fat percentage; fat pad mass; forced expiratory volume; Inflammation/hematologic traits; Lifestyle/behavioral traits; Lipid/fatty acid; Liver-related traits; Metabolic/endocrine dysfunction; protein measurement; serum metabolite level; vital capacity
rs7133378	adiposity, body fat distribution, cardiometabolic, lipid-related, endocrine, inflammatory, and hematologic traits
rs7138803	age at menarche; Inflammation/hematologic traits; Lifestyle/behavioral traits; Lipid/fatty acid; Metabolic/endocrine dysfunction; waist circumference; waist-hip ratio
rs7141420	base metabolic rate measurement; body height; lean body mass; Lifestyle/behavioral traits; whole body water mass
rs715	a broad range of metabolic and renal traits
rs7164727	body fat percentage; Lifestyle/behavioral traits; Lipid/fatty acid; Metabolic/endocrine dysfunction; waist circumference
rs7186893	Metabolic/endocrine dysfunction; waist-hip ratio
rs7195386	body height
rs7200589	Blood pressure; body height
rs7239114	comparative body size at age 10, self-reported; Metabolic/endocrine dysfunction
rs72779695	age at first birth measurement; Educational/socioeconomic status
rs738140	body height
rs7498044	body height
rs7498665	body fat percentage; body height; fat pad mass; Liver-related traits; Neurological/psychiatric dysfunction; visceral adipose tissue quantity; waist circumference; waist-hip ratio
rs750090	taste liking measurement
rs7550711	Lifestyle/behavioral traits; Lipid/fatty acid; visceral adipose tissue quantity; waist circumference
rs756717	hip circumference
rs7588437	Metabolic/endocrine dysfunction
rs7599312	Lifestyle/behavioral traits; Metabolic/endocrine dysfunction; waist-hip ratio
rs7621025	cardiovascular and coagulation-related traits
rs768023	Liver-related traits; Metabolic/endocrine dysfunction; Neurological/psychiatric dysfunction; sexual dimorphism measurement
rs7713317	body height; Metabolic/endocrine dysfunction
rs78886584	body height
rs7899106	Metabolic/endocrine dysfunction
rs7961979	Metabolic/endocrine dysfunction
rs8075273	waist-hip ratio
rs811054	body fat percentage; body height; hip circumference; Lipid/fatty acid; Metabolic/endocrine dysfunction
rs879620	adiposity, body composition, metabolic, inflammatory, lipid-related, liver-related, musculoskeletal, and renal traits
rs901630	Liver-related traits; Metabolic/endocrine dysfunction; Neurological/psychiatric dysfunction; waist-hip ratio
rs905938	body composition, anthropometric, and reproductive traits

rs925421	Lifestyle/behavioral traits
rs9395747	Metabolic/endocrine dysfunction
rs942066	asthma, irritable bowel syndrome
rs943005	Lifestyle/behavioral traits; waist circumference
rs947088	comparative body size at age 10, self-reported
rs947791	lean body mass
rs9540493	Metabolic/endocrine dysfunction
rs9906944	body fat percentage; coronary artery disease; Metabolic/endocrine dysfunction; Neurological/psychiatric dysfunction; verbal-numerical reasoning measurement
rs9937053	Cardiometabolic traits; Lifestyle/behavioral traits
rs9955276	body fat percentage; Lifestyle/behavioral traits

Supplementary Table 11 Baseline characteristics between individuals with and without PMDs, among individuals included in one-sample MR in LifeGene

	Individuals without PMDs	Individuals with PMDs
Total number	4,945	1,058
Age	34.0±8.5	34.5±8.1
Age at menarche	12.9±1.4	12.8±1.4
BMI[a]	22.8±3.0	23.1±3.2
METs	13.2±20.3	13.3±19.4
Income		
<= Q1	1,192 (24.1)	308 (29.1)
>Q1 and <Q3	2,480 (50.2)	523 (49.4)
≥Q3	1,273 (25.7)	227 (21.5)
Civil status		
Non-cohabitated	1,383 (28.0)	258 (24.4)
Cohabitated	3,562 (72.0)	800 (75.6)
Education level		
High school or below	909 (18.4)	184 (17.4)
University	3,557 (71.9)	747 (70.6)
Postgraduate	479 (9.7)	127 (12.0)
Country of birth		
Sweden	4,632 (93.7)	940 (88.8)
Outside Sweden	313 (6.3)	118 (11.2)
Place of residence		
Stockholm county	3,971 (80.3)	873 (82.5)
Other counties	974 (19.7)	185 (17.5)
Smoking		
<100 cigarettes in life	2,923 (59.1)	498 (47.1)
≥100 cigarettes in life	1,972 (39.9)	544 (51.4)
Unknown	50 (1.0)	16 (1.5)
Childhood abuse		
No	3,317 (67.1)	567 (53.6)
Yes	1,628 (32.9)	491 (46.4)
Alcohol drinking		
Never	163 (3.3)	41 (3.9)
1-3 times a month or less often	2,509 (50.7)	522 (49.3)
More than once a week	2,273 (46.0)	495 (46.8)
Parity		
0	2,962 (59.9)	617 (58.3)
1+	1,983 (40.1)	441 (41.7)
Depression		
No	4,318 (87.3)	734 (69.4)
Yes	627 (12.7)	324 (30.6)
Anxiety		

No	4,363 (88.2)	820 (77.5)
Yes	582 (11.8)	238 (22.5)
Use of oral contraceptives		
No	4,631 (93.7)	991 (93.7)
Yes	314 (6.3)	67 (6.3)

BMI, body mass index; METs, metabolic equivalents of task; PMDs, premenstrual disorder.
[a]For a small proportion of individuals (n=94), BMI was extracted from MBR if missing in baseline survey in LifeGene.
Covariates with few missing(<5%) were imputed to the most common level. Missing values in age (n=59), age at menarche (n=329), BMI (n=209) was not shown.

Supplementary Table 12 Assessment of instrument strength for smoking and age at menarche in one-sample MR

	**Beta/OR**	**PRS R2 (%)**	**F statistics**
**Age at menarche**	0.44 (0.40-0.48)	9.4	589
**BMI**	0.32 (0.24-0.39)	1.1	64
**Smoking**	1.14 (1.08-1.21)	0.5	32

BMI, body mass index; OR, odds ratio; PRS, polygenic risk score.

We calculated pseudo-R2 using the Nagelkerke method for smoking. F staitiscs was calculated by the formula $F = \frac{(n-k-1)}{k}\frac{R2}{(1-R2)}$, where R2 is the variance in the exposure explanied by PRS, n is the sample size and k is the number of instruments.

Supplementary Table 13 Power calculation

Exposure	OR[a]	Sample size	Ratio of control:case	Statistical power (%)
Two-sample MR				
Age at menarche	0,93	72,297	3	95
Anemia	1,13	72,297	3	17
BMI	1,03	72,297	3	13
Childhood abuse	1,2-1,8	72,297	3	10-58
Childhood asthma	1,15	72,297	3	100
Diabetes	1,68	72,297	3	100
Endometriosis	1,52	72,297	3	100
Smoking	2,1	72,297	3	100
One-sample MR				
Age at menarche	0,94	5,674	5	8
BMI	1,19	5,794	5	6
Smoking	1,25	5,937	5	7

BMI, body mass index; MR, Mendelian Randomization; OR, odds ratio.
Statistical power was calulcated based on https://sb452.shinyapps.io/power/. R^2 explained by genetic variants was derived from Table S3 in two-sample MR and Table S7 in one-sample MR.
[a]For two-sample MR, we used OR reported from prospective studies. If the original study reported RR/HR and the prevalence of PMDs is above 10%, we converted them to OR. For one-sample MR, we did *posthoc* power calculation and used OR from two-sample MR, since we consider OR from two-sample MR likely represent the true causal estimate.

Supplementary table 14 Sensitivity analysis using female-specific smoking GWAS

A. Association between genetically liability to smoking and PMDs in two-sample MR

method	nsnp	OR	P
MR Egger	21	0.35 (0.04-3.04)	0.351
Weighted median	21	1.13 (0.89-1.42)	0.315
Inverse variance weighted	21	1.21 (0.97-1.49)	0.085
Weighted mode	21	1.06 (0.61-1.83)	0.832

The F statistics was 157, indicating a strong instrument. Although Cochran's Q and MR-PRESSO global test suggested pleiotropy among SNPs ($p<0.05$), the MR-Egger test did not indicate directional pleiotropy (p for intercept > 0.05).

B. Association between genetically predicted liability to smoking and PMDs in one-sample MR in LifeGene

nsnp	OR	P
33	1.07 (0.86-1.33)	0.539

The smoking PRS was positively associated with smoking phenotype in LifeGene (OR=1.13 (1.07-1.19), $p<0.001$).

Supplementary Table 15 IVW, MR Egger, Weighted median and weighted mode regression for subtypes of PMDs

Exposure	Outcome	Method	N snp	OR	P
Smoking	PMDD	MR Egger	81	1.77 (0.45-1.33)	0.416
Smoking	PMDD	Weighted median	81	1.06 (0.73-1.03)	0.768
Smoking	PMDD	IVW	81	0.92 (0.73-0.96)	0.491
Smoking	PMDD	Weighted mode	81	1.91 (0.75-1.38)	0.178
Smoking	With depression or anxiety	MR Egger	80	1.78 (0.24-1.33)	0.566
Smoking	With depression or anxiety	Weighted median	80	1.18 (0.69-1.09)	0.534
Smoking	With depression or anxiety	IVW	80	0.97 (0.69-0.99)	0.875
Smoking	With depression or anxiety	Weighted mode	80	2.43 (0.66-1.56)	0.186
Smoking	Without depression and anxiety	MR Egger	81	1.55 (0.35-1.24)	0.573
Smoking	Without depression and anxiety	Weighted median	81	1.24 (0.82-1.11)	0.304
Smoking	Without depression and anxiety	IVW	81	0.98 (0.77-0.99)	0.917
Smoking	Without depression and anxiety	Weighted mode	81	1.91 (0.75-1.38)	0.174
BMI	PMDD	MR Egger	238	0.90 (0.10-8.30)	0.923
BMI	PMDD	Weighted median	238	1.37 (0.70-2.69)	0.360
BMI	PMDD	IVW	238	1.39 (0.85-2.25)	0.186
BMI	PMDD	Weighted mode	238	1.15 (0.14-9.72)	0.898
BMI	With depression or anxiety	MR Egger	241	0.71 (0.03-14.73)	0.826
BMI	With depression or anxiety	Weighted median	241	1.71 (0.65-4.47)	0.276
BMI	With depression or anxiety	IVW	241	1.79 (0.92-3.48)	0.086
BMI	With depression or anxiety	Weighted mode	241	0.67 (0.03-14.72)	0.799
BMI	Without depression and anxiety	MR Egger	240	1.28 (0.12-13.81)	0.841
BMI	Without depression and anxiety	Weighted median	240	1.10 (0.55-2.21)	0.778
BMI	Without depression and anxiety	IVW	240	0.97 (0.60-1.58)	0.910

BMI	Without depression and anxiety	Weighted mode	240	1.32 (0.16-10.77)	0.794
Age at menarche	PMDD	MR Egger	769	1.19 (0.78-1.82)	0.412
Age at menarche	PMDD	Weighted median	769	1.02 (0.78-1.34)	0.863
Age at menarche	PMDD	IVW	769	0.97 (0.83-1.15)	0.740
Age at menarche	PMDD	Weighted mode	769	1.02 (0.60-1.73)	0.937
Age at menarche	With depression or anxiety	MR Egger	771	0.93 (0.51-1.70)	0.809
Age at menarche	With depression or anxiety	Weighted median	771	0.80 (0.54-1.17)	0.249
Age at menarche	With depression or anxiety	IVW	771	0.91 (0.72-1.15)	0.438
Age at menarche	With depression or anxiety	Weighted mode	771	1.15 (0.49-2.69)	0.746
Age at menarche	Without depression and anxiety	MR Egger	770	1.33 (0.84-2.12)	0.221
Age at menarche	Without depression and anxiety	Weighted median	770	1.20 (0.88-1.62)	0.253
Age at menarche	Without depression and anxiety	IVW	770	1.02 (0.85-1.21)	0.865
Age at menarche	Without depression and anxiety	Weighted mode	770	1.30 (0.74-2.29)	0.367

BMI, body mass index; IVW, inverse-variance weighted; PMDD, premenstrual dysphoric disorder.
Association of each SNP with AAM, BMI and smoking comes from external datasets. Association between each SNP and subtypes of PMDs were estimated in LifeGene using plink, and adjusted for age at baseline, first 10 principal components and substudy membership. Analyses for PMS was not performed due to few cases. The OR for smoking refers to OR per SD increase in standardized predicted probability of smoking, and ORs for AAM and BMI refer to OR per unit (year for AAM and kg/m^2 for BMI) increase in their predicted values.

Supplementary Figure 1 Phenotypic associations of age at menarche, BMI and smoking with PMDs and its subtypes, among individuals included in one-sample MR in LifeGene

Exposure	Outcome	N	OR (95% CI)	P
AAM	PMDs	5,674	0.93 (0.88-0.98)	0.004
AAM	Severe PMS	5,372	0.91 (0.77-1.08)	0.284
AAM	Probable PMDD	5,372	0.93 (0.89-0.99)	0.012
AAM	With depression or anxiety	5,674	1.00 (0.91-1.09)	0.911
AAM	Without depression and anxiety	5,674	0.89 (0.84-0.95)	<0.001
AAM ≤1 SD	PMDs	5,674	1.34 (1.11-1.62)	0.003
AAM ≥1 SD	PMDs	5,674	0.90 (0.72-1.13)	0.370
AAM ≤1 SD	Severe PMS	5,372	1.32 (0.71-2.47)	0.383
AAM ≥1 SD	Severe PMS	5,372	0.79 (0.35-1.76)	0.563
AAM ≤1 SD	Probable PMDD	5,372	1.28 (1.05-1.56)	0.016
AAM ≥1 SD	Probable PMDD	5,372	0.93 (0.73-1.17)	0.521
AAM ≤1 SD	With depression or anxiety	5,674	1.13 (0.81-1.58)	0.469
AAM ≥1 SD	With depression or anxiety	5,674	1.05 (0.72-1.55)	0.791
AAM ≤1 SD	Without depression and anxiety	5,674	1.48 (1.17-1.86)	0.001
AAM ≥1 SD	Without depression and anxiety	5,674	0.84 (0.64-1.10)	0.205
BMI	PMDs	5,794	1.01 (0.99-1.04)	0.278
BMI	Severe PMS	5,495	1.03 (0.95-1.11)	0.502
BMI	Probable PMDD	5,495	1.01 (0.99-1.04)	0.312
BMI	With depression or anxiety	5,794	1.04 (1.00-1.08)	0.045
BMI	Without depression and anxiety	5,794	1.00 (0.97-1.03)	0.951
Underweight	PMDs	5,794	0.65 (0.38-1.12)	0.121
Overweight	PMDs	5,794	0.94 (0.78-1.12)	0.486
Obese	PMDs	5,794	1.21 (0.85-1.73)	0.292
Underweight	Severe PMS	5,495	1.28 (0.30-5.41)	0.735
Overweight	Severe PMS	5,495	0.61 (0.30-1.26)	0.182
Obese	Severe PMS	5,495	1.58 (0.55-4.57)	0.397
Underweight	Probable PMDD	5,495	0.66 (0.37-1.15)	0.144
Overweight	Probable PMDD	5,495	0.97 (0.80-1.17)	0.751
Obese	Probable PMDD	5,495	1.15 (0.79-1.69)	0.463
Underweight	With depression or anxiety	5,794	0.47 (0.18-1.19)	0.110
Overweight	With depression or anxiety	5,794	1.02 (0.75-1.38)	0.918
Obese	With depression or anxiety	5,794	1.32 (0.73-2.40)	0.363
Underweight	Without depression and anxiety	5,794	0.89 (0.47-1.70)	0.727
Overweight	Without depression and anxiety	5,794	0.91 (0.73-1.14)	0.414
Obese	Without depression and anxiety	5,794	1.17 (0.75-1.85)	0.487
Smoking	PMDs	5,937	1.42 (1.23-1.64)	<0.001
Smoking	Severe PMS	5,625	0.82 (0.48-1.38)	0.445
Smoking	Probable PMDD	5,625	1.50 (1.29-1.74)	<0.001
Smoking	With depression or anxiety	5,937	1.34 (1.04-1.72)	0.022
Smoking	Without depression and anxiety	5,937	1.49 (1.25-1.78)	<0.001

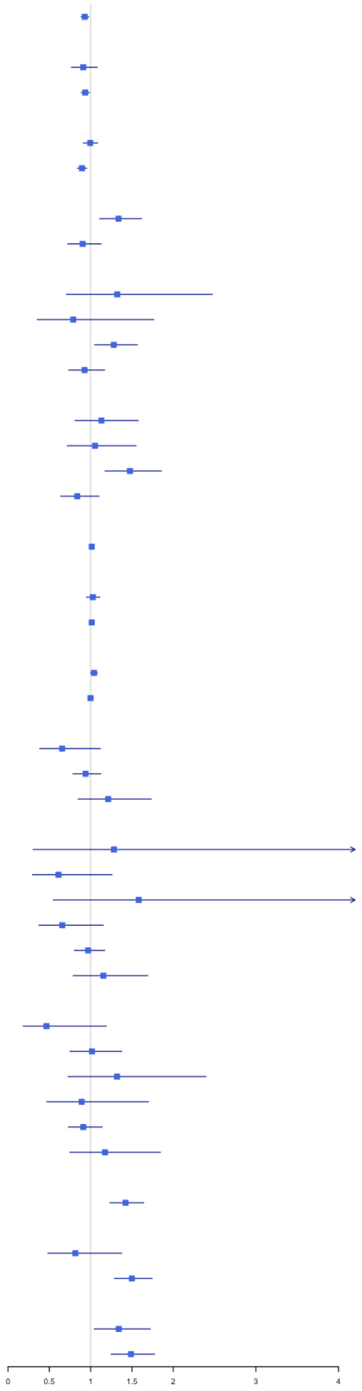

AAM, age at menarche; BMI, body mass index.
OR was estimated using logistic regression, and adjusted for age, income, civil status, country of birth, place of residence, education level, BMI, smoking, alcohol drinking, parity, age at menarche, physical activity, childhood abuse, depression, anxiety and use of OC. Analyses on category of age at menarche used individuals whose age at menarche is between mean ± 1sd as the reference group. Analyses on category of BMI used individuals who had normal BMI as the reference group.

Supplementary Figure 2 Causal associations of age at menarche, BMI and smoking with PMDs in one-sample MR in LifeGene, by different ascertainment approach of PMDs

Exposure	Source of PMDs	N individual	N case	N SNP	OR (95% CI)	P
Age at menarche	LifeGene	5,372	962	772	0.99 (0.83-1.17)	0.866
Age at menarche	Clinical diagnoses	5,674	159	772	0.88 (0.61-1.26)	0.485
BMI	LifeGene	5,495	964	241	1.13 (0.90-1.42)	0.309
BMI	Clinical diagnoses	5,794	161	241	1.60 (0.97-2.64)	0.066
Smoking	LifeGene	5,625	989	103	0.91 (0.73-1.12)	0.366
Smoking	Clinical diagnoses	5,937	161	103	1.02 (0.65-1.61)	0.924

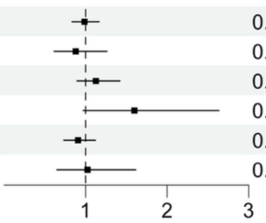

BMI, body mass index; CI, confidence interval; N SNPs, number of single nucleotide polymorphisms; OR, odds ratio.
Analyses were performed on unrelated individuals with European ancestry who aged 16-60 at enrollment, had menstruated in the past year, and had genotype and exposure data. The ORs for smoking refer to OR per SD increase in standardized predicted probability of smoking, and ORs for AAM and BMI refer to OR per unit (year for AAM and kg/m^2 for BMI) increase in their predicted values.

Reference

1. Toivonen J, Allara E, FinnGen, Castrén J, di Angelantonio E, Arvas M. The value of genetic data from 665,460 individuals in managing iron deficiency anaemia and suitability to donate blood. *Vox Sang*. 2024;119(1):34-42. doi:10.1111/vox.13564

2. Kentistou KA, Kaisinger LR, Stankovic S, et al. Understanding the genetic complexity of puberty timing across the allele frequency spectrum. *Nat Genet*. 2024;56(7):1397-1411. doi:10.1038/s41588-024-01798-4

3. Pulit SL, Stoneman C, Morris AP, et al. Meta-analysis of genome-wide association studies for body fat distribution in 694 649 individuals of European ancestry. *Hum Mol Genet*. 2019;28(1):166-174. doi:10.1093/hmg/ddy327

4. Sakaue S, Kanai M, Tanigawa Y, et al. A cross-population atlas of genetic associations for 220 human phenotypes. *Nat Genet*. 2021;53(10):1415-1424. doi:10.1038/s41588-021-00931-x

5. Chen TT, Chen CY, Liu CY, et al. Genetic architectures of childhood maltreatment and causal influence of childhood maltreatment on health outcomes in adulthood. *Mol Psychiatry*. 2025;30(8):3404-3412. doi:10.1038/s41380-025-02928-y

6. Mahajan A, Taliun D, Thurner M, et al. Fine-mapping type 2 diabetes loci to single-variant resolution using high-density imputation and islet-specific epigenome maps. *Nat Genet*. 2018;50(11):1505-1513. doi:10.1038/s41588-018-0241-6

7. Pujol Gualdo N, Džigurski J, Rukins V, et al. Atlas of genetic and phenotypic associations across 42 female reproductive health diagnoses. *Nat Med*. 2025;31(5):1626-1634. doi:10.1038/s41591-025-03543-8

8. Liu M, Jiang Y, Wedow R, et al. Association studies of up to 1.2 million individuals yield new insights into the genetic etiology of tobacco and alcohol use. *Nat Genet*. 2019;51(2):237-244. doi:10.1038/s41588-018-0307-5
